# Supplementary figures and images for: Evolution of a research field—a micro (RNA) example
Source: PeerJ. 2015 Mar 17;3:e829. doi: 10.7717/peerj.829 (PMC4369334; doi:10.7717/peerj.829)

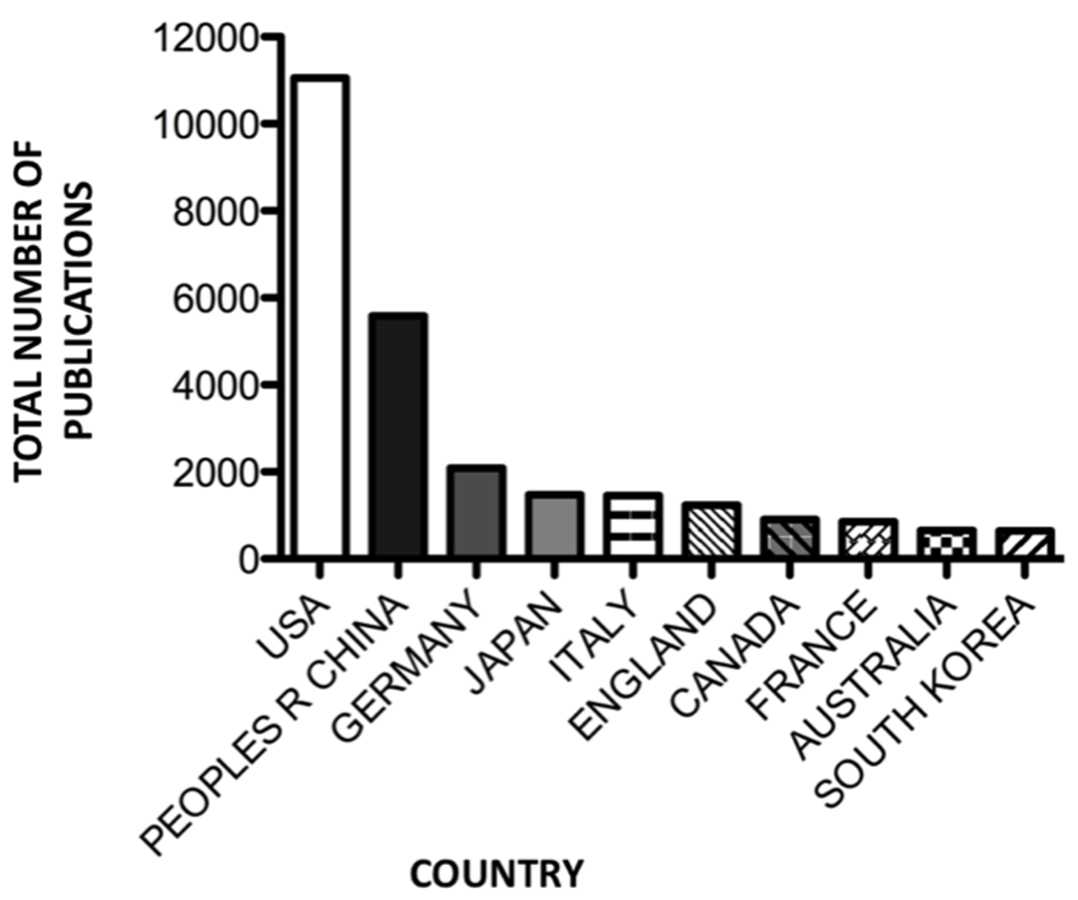

Supplement: Figure S1 — Rankings adjusted from Figure 3 due to the United Kingdom being separated according to the individual constituent countries: England, Wales, Scotland and Northern Ireland. [file peerj-03-829-s001.png]
