# Supplementary material for: Evolution of a research field—a micro (RNA) example
Source: PeerJ. 2015 Mar 17;3:e829. doi: 10.7717/peerj.829 (PMC4369334; doi:10.7717/peerj.829)
Supplement: Table S1 [file peerj-03-829-s002.docx]

| **RANK** | **JOURNAL** | **NO. PUBLICATIONS** | **RANK** | **JOURNAL** | **NO. PUBLICATIONS** |
| --- | --- | --- | --- | --- | --- |
| 1 | PLoS ONE | 1589 | 26 | Circulation Research | 135 |
| 2 | Nucleic Acids Research | 489 | 27 | Clinical Cancer Research | 134 |
| 3 | PNAS | 451 | 28 | Molecular Cell | 125 |
| 4 | BLOOD | 432 | 29 | Gene | 123 |
| 5 | Journal of Biological Chemistry | 346 | 30 | Carcinogenesis | 120 |
| 6 | Biochem Biophys Res Communications | 329 | 31 | British Journal of Cancer | 116 |
| 7 | RNA a Publication of the RNA Society | 303 | 32 | International Journal of Molecular Sciences | 114 |
| 8 | Cancer Research | 302 | 33 | Bioinformatics | 109 |
| 9 | BMC Genomics | 296 | 34 | Journal of Immunology | 107 |
| 10 | Hepatology | 252 | 35 | BMC Bioinformatics | 102 |
| 11 | Circulation | 246 | 36 | Plant Cell | 102 |
| 12 | Oncogene | 211 | 37 | International Journal of Oncology | 101 |
| 13 | Faseb Journal | 210 | 38 | Neuro Oncology | 99 |
| 14 | Cell Cycle | 200 | 39 | Molecular Biology Reports | 97 |
| 15 | Febs Letter | 180 | 40 | Science | 96 |
| 16 | Modern Pathology | 179 | 41 | European Journal of Cancer | 95 |
| 17 | Gastroenterology | 176 | 42 | Febs Journal | 93 |
| 18 | Laboratory Investigation | 171 | 43 | Molecular Therapy | 93 |
| 19 | Genes Development | 167 | 44 | Genome Research | 92 |
| 20 | Journal of Virology | 162 | 45 | Development | 91 |
| 21 | Cell | 159 | 46 | American J Respir Critical Care Med | 90 |
| 22 | RNA Biology | 146 | 47 | Current Biology | 90 |
| 23 | International Journal of Cancer | 136 | 48 | EMBO Journal | 90 |
| 24 | Nature | 136 | 49 | PLoS Genetics | 90 |
| 25 | Oncology Reports | 136 | 50 | Cancer Letters | 89 |
