# Supplementary material for: Evolution of a research field—a micro (RNA) example
Source: PeerJ. 2015 Mar 17;3:e829. doi: 10.7717/peerj.829 (PMC4369334; doi:10.7717/peerj.829)
Supplement: Table S2 [file peerj-03-829-s003.docx]

| **RANK** | **JOURNAL** | **ARTICLES** | **REVIEWS** | **CORRECTIONS** | **EDITORIAL MATERIAL** | **LETTER** | **MEETING ABSTRACT** | **PROCEEDINGS PAPERS** | **DATABASE REVIEWS** |
| --- | --- | --- | --- | --- | --- | --- | --- | --- | --- |
| 1 | PLOS ONE | 1587 | 2 | 2 | 2 | - | - | - | - |
| 2 | Nucleic Acids Research | 485 | 3 | 3 | 3 | - | - | - | - |
| 3 | PNAS | 438 | - | - | - | 1 | - | - | - |
| 4 | Blood | 170 | 13 | 13 | 13 | 1 | 239 | 1 |  |
| 5 | Journal of Biological Chemistry | 315 | 6 | 6 | 6 | - | - | 1 | - |
| 6 | Biochem Biophysical Research Comm | 310 | 17 | 17 | 17 | - | - | - | - |
| 7 | RNA a Publication of the RNA Society | 283 | 13 | 13 | 13 | 2 | - | - | - |
| 8 | Cancer Research | 267 | 17 | 17 | 17 | 2 | 10 | - | - |
| 9 | BMC Genomics | 293 | - | - |  |  |  | 19 | 1 |
| 10 | Hepatology | 93 | 2 | 2 | 2 | 11 | 144 | - | - |
