# Supplementary material for: Evolution of a research field—a micro (RNA) example
Source: PeerJ. 2015 Mar 17;3:e829. doi: 10.7717/peerj.829 (PMC4369334; doi:10.7717/peerj.829)
Supplement: Table S3 [file peerj-03-829-s004.docx]

| **RANK** | **COUNTRY** | **NO. PUBLICATIONS** | **NO. CITATIONS** | **AVERAGE CITATION PER ITEM** |
| --- | --- | --- | --- | --- |
| 1 | United States | 11056 | 475,300 | 44.3 |
| 2 | Peoples Republic of China | 5584 | 72,265 | 14.5 |
| 3 | Germany | 2083 | 71,051 | 35.44 |
| 4 | Japan | 1474 | 41,218 | 29.66 |
| 5 | Italy | 1455 | 47,084 | 34.19 |
| 6 | England | 1239 | 42,970 | 35.9 |
| 7 | Canada | 904 | 24,627 | 27.6 |
| 8 | France | 848 | 28,875 | 35.37 |
| 9 | Australia | 650 | 17,414 | 28.4 |
| 10 | South Korea | 645 | 17,094 | 27.9 |
| 11 | Netherlands | 625 | 25,281 | 39.63 |
| 12 | Spain | 621 | 14,816 | 23.44 |
| 13 | Switzerland | 492 | 27,450 | 55.12 |
| 14 | Denmark | 420 | 15,162 | 35.34 |
| 15 | Taiwan | 411 | 8,151 | 19.64 |
| 16 | India | 371 | 5,373 | 14.52 |
| 17 | Israel | 348 | 12,592 | 35.27 |
| 18 | Sweden | 335 | 12,238 | 35.78 |
| 19 | Belgium | 292 | 7,491 | 25.31 |
| 20 | Ireland | 254 | 5,108 | 20.11 |
