# Supplementary material for: Evolution of a research field—a micro (RNA) example
Source: PeerJ. 2015 Mar 17;3:e829. doi: 10.7717/peerj.829 (PMC4369334; doi:10.7717/peerj.829)
Supplement: Table S4 [file peerj-03-829-s005.docx]

| **RANK** | **CITATIONS** | **TITLE** | **LEAD AUTHOR** | **JOURNAL** | **YEAR** |
| --- | --- | --- | --- | --- | --- |
| 1 | 4,167 | Conserved seed pairing, often flanked by adenosines, indicates that thousands of human genes are microRNA targets | Lewis BP | Cell | 2005 |
| 2 | 3,743 | MicroRNAs: Target Recognition and Regulatory Functions | Bartel DP | Cell | 2009 |
| 3 | 3,671 | The C-Elegans heterochronic gene Lin-4 encodes small RNAs with antisense complementarity to Lin-14 | Lee RC | Cell | 1993 |
| 4 | 3,512 | MicroRNA expression profiles classify human cancers | Lu J | Nature | 2005 |
| 5 | 2,976 | The functions of animal microRNAs | Ambros V. | Nature | 2004 |
| 6 | 2,391 | Oncomirs - microRNAs with a role in cancer | Esquela-Derscher A | Nature Reviews Cancer | 2006 |
| 7 | 2,337 | Mapping and quantifying mammalian transcriptomes by RNA-Seq | Mortazavi A | Nature Methods | 2008 |
| 8 | 2,291 | A microRNA expression signature of human solid tumors defines cancer gene targets | Volinia S | PNAS | 2006 |
| 9 | 2,196 | MicroRNA signatures in human cancers | Calin GA | Nature Reviews Cancer | 2006 |
| 10 | 2,168 | Microarray analysis shows that some microRNAs downregulate large numbers of target mRNAs | Lim LP | Nature | 2005 |
